# The involvement and significance of M2 macrophages in neuropathic pain following spinal cord injury: a systematic review

**DOI:** 10.1186/s12576-024-00932-5

**Published:** 2024-09-18

**Authors:** Aidin Shahrezaei, Maryam Sohani, Mohammadhassan Sohouli, Soroush Taherkhani, Farinaz Nasirinezhad

**Affiliations:** 1 School of Medicine, Iran University of Medical Sciences, Tehran, Iran; 2 Student Research Committee, Department of Clinical Nutrition and Dietetics, Faculty of Nutrition and Food Technology, Shahid Beheshti University of Medical Sciences, Tehran, Iran; 3 Department of Physiology, Iran University of Medical Sciences, Tehran, Iran; 4 Physiology Research Center, Iran University of Medical Sciences, Tehran, Iran; 5 Center of Experimental and Comparative Study, Iran University of Medical Sciences, Tehran, Iran

**Keywords:** Neuropathic pain, Spinal cord, Injury, Macrophage, Anti-inflammatory, Allodynia

## Abstract

Neuropathic pain (NeP) is a type of persistent pain initiated by diseases or injuries of the nervous system. Although the underlying pathophysiological mechanisms of NeP are poorly understood, the immune system plays a key role in this condition. M2 macrophages have a key role in tissue healing and the reduction of inflammation. This systematic study aims to provide an overview of the role and importance of M2 macrophages in NeP after spinal cord injury (SCI). A comprehensive systematic review was conducted utilizing Scopus, PubMed, Embase, and ISI Web of Science databases. Two independent reviewers conducted the article selection. All publications examine the impact of M2 macrophages on NeP following spinal cord injuries. A quality assessment was conducted on bias entities that had been predetermined. Eleven papers met the criteria. According to the findings, focusing on immune cell polarization presents viable therapeutic options for treating NeP and enhancing recovery after SCI. M2 macrophages are essential for reducing neuropathic pain and promoting recovery after spinal cord injury. The modulation of M2 macrophages by a number of therapeutic approaches, including ivermectin-functionalized MWCNTs, isorhamnetin, Neuregulin-1 administration, TMEM16F inhibition, lentivirus-mediated delivery of anti-inflammatory cytokines, epigallocatechin-3-gallate, and red-light therapy promotes neuroregeneration, decreases neuroinflammatory cytokines, and reduces NeP. The results of these preclinical investigations must, however, be interpreted with caution, according to the quality assessment and risk of bias analysis of the studies that were included. Targeting M2 macrophages may have therapeutic benefits as they are essential for the management of NeP and recovery following spinal cord damage.

**Supplementary Information:**

The online version contains supplementary material available at 10.1186/s12576-024-00932-5.

## Introduction

Neuropathic pain (NeP) is pain that occurs due to structural or functional issues with the nervous system and commonly found in some diseases related to the central nervous system (CNS) [1, 2]. NeP is a complex and chronic pain condition affecting 7–10% of the global population [3]. It has a significant impact on both physical and functional abilities, mental well-being, and daily activities [4, 5]. Despite its long-lasting nature, NeP is an intricate condition with temporal variations [6]. Common treatments for NeP include antidepressant drugs, anticonvulsant medications, and lifestyle treatments such as physical, relaxation, and massage therapies. However, complications may arise from side effects of medications and the need for ongoing management to prevent symptoms worsening and maintain daily functioning is absolutely necessary [7, 8].

Numerous scientific investigations about the pathophysiology of NeP have demonstrated that the immune cells, particularly macrophages, play a critical role in the pathogenesis of neuropathic pain [9]. In the context of development of NeP following spinal cord injury (SCI), the interplay between infiltrating macrophages, activated microglial cells, and damage to the blood–spinal cord barrier (BSCB) has been suggested [6, 10].

About the mechanisms involved in the initiation and maintenance of Neuropathic pain the function of macrophages has garnered significant research interest due to the two subclasses of the cells: the classically activated macrophages (M1 phenotype) and the alternatively activated macrophages (M2 phenotype) [11, 12]. The activation of M1 phenotype, a type of macrophage, is induced by exposure to T helper 1 (Th1) cytokines, interferon-γ (IFN-γ) and tumor necrosis factor-α (TNF-α). In contrast, the M2 phenotype, a distinct subclass of macrophages from M1, is stimulated by T helper 2 (Th2) cytokines, interleukin (IL)-4, IL-10, transforming growth factor-beta (TGF-beta), and nerve growth factor (NGF) [11, 13]. M2 macrophages play a critical role in promoting regenerative growth and exerting an anti-inflammatory response within spinal cord [14, 15]. In contrast, the M1 phenotype’s conspicuous production of inflammatory cytokines is widely believed to be a contributing factor to NeP [12]. However, the M2 phenotype's superior ability to modulating the inflammatory response may contribute to pain alleviation [16].

Research suggests that the analgesic properties of M2 macrophages is facilitated by opioid pathways, and a decrease in the M1/M2 ratio contributes to the alleviation of neuropathic pain [17–19]. However, the effect of these macrophages on neuropathic pain has not been comprehensively studied yet.

Therefore, this systematic review examines the involvement and significance of M2 macrophages in NeP that occurs after SCI.

## Methods

### Design

This study followed the Preferred Reporting Items for Systematic Reviews and Meta-Analyses (PRISMA) guidelines.

### Search strategy

We systematically searched the following original electronic databases from inception to 16 June 2023: PubMed/Medline, Web of Science, Embase, and Scopus to identify all articles on the role of M2 macrophages in neuropathic pain following spinal cord injury. The following terms were searched as all fields, and MeSH terms: Macrophage, “Epithelioid Cell", Histiocytes, monocyte, “Foam cell”, “Foreign body giant cell”, “langhans giant cell”, Neuralgia, Causalgia, Neuralgia, "Neuropathic Pain", neutropenia, "Nerve Pain", Hyperalgesia, Allodynia, Sciatica, “Spinal Cord Injury”, “Central Cord Syndrome”, “Autonomic Dysreflexia”, “Spinal Cord Compression”, “Spinal Fracture”, “Spinal Cord Trauma”, “Traumatic Myelopathy”, " Spinal Cord Laceration ", “Post-Traumatic Myelopathy”, “Spinal Cord Contusion” [Supplementary Material 1]. Apart from the above, no publication date or language restrictions were applied. After removal of duplicates two independent reviewers, screened the titles and abstracts of articles retrieved by the initial search, and thereafter at the full-text level. We also checked relevant articles such as review articles for additional references. Differences in opinion were resolved by consensus and when needed a third reviewer was consulted.

In this systematic review, we included all controlled animal experiments that assessed the involvement and significance of M2 macrophages in ameliorating neuropathic pain following spinal cord injury. Inclusion criteria were as follows: (1) original nonhuman studies that have investigated neuropathic pain in the context of SCI; (2) studies that have specifically manipulated or analyzed M2 macrophages or its related cytokines in relation to neuropathic pain; (3) control interventions consisted of placebo (saline, culture medium, or similar vehicle) or no treatment; (4) studies that have assessed neuropathic pain using valid and reliable pain measurement tools. Exclusion criteria included: (1) studies that lack a control group, case reports, case series, review articles, conference abstracts; (2) the neuropathic pain secondary to other disease models, central nerve system diseases, or diabetic neuropathy; (4) studies that focus on other types of immune cells or molecular pathways, or those that do not differentiate between M1 and M2 macrophages; (5) studies that only report outcomes unrelated to pain, such as motor function or inflammation, and do not provide quantitative data on pain or M2 macrophage levels; (6) studies with insufficient quantitative data to extract threshold values were excluded.

### Data extraction and tabulation

The following data were extracted and recorded in Table 1: general information (authors, publication year), demographic data (animal number per group, control group, age, sex, species, and strain), treatment protocol (drug, transplantation cell or light therapy dosage/wave lengths, duration of administration and study), tests and assessments and outcomes.

**Table 1:** Preclinical study characteristics summary

Number	Author name/YEAR	Species	Strain	Age/ Weight	Genetic Background	Number of ANIMALS per group	Definition of Control	Method of Allocation to Treatments	Target Tissue	Using Appropriate Tests	Blindness of Assessor	Randomization	Definition of the experimental unit (individual animal/animals in one cage)	Description of Statistical	animal facility	Ethics	Description of the Reasons to Exclude Animals from the Experiment during the Study
1	Dexiang Ban/2022	Yes	Yes	Yes	Yes	Yes	Yes	Yes	Yes	Yes	Yes	Yes	Yes	Yes	Yes	Yes	N/R
2	Alireza Rahbar/2021	Yes	Yes	Yes	Yes	N/R	Yes	Yes	Yes	Yes	Yes	N/R	N/R	Yes	Yes	Yes	N/R
3	Fei Chen/2021	Yes	Yes	Yes	Yes	Yes	Yes	Yes	Yes	Yes	Yes	N/R	N/R	Yes	Yes	Yes	N/R
4	Hideaki Nakajima/2020	Yes	Yes	Yes	Yes	Yes	N/R	Yes	Yes	Yes	N/R	Yes	N/R	Yes	N/R	Yes	N/R
5	Jing Zhao/2019	Yes	Yes	N/R	Yes	N/R	Yes	Yes	Yes	Yes	Yes	N/R	N/R	Yes	N/R	no	N/R
6	Jonghyuck Park/ 2018	Yes	Yes	Yes	Yes	N/R	Yes	Yes	Yes	Yes	Yes	Yes	N/R	Yes	N/R	Yes	N/R
7	Arsalan Alizadeh/2018	Yes	Yes	Yes	Yes	Yes	Yes	Yes	Yes	Yes	Yes	Yes	N/R	Yes	Yes	Yes	N/R
8	Lucia Machova Urdzikova/2017	Yes	Yes	Yes	Yes	Yes	Yes	Yes	Yes	Yes	N/R	Yes	N/R	Yes	Yes	Yes	N/R
9	Di Hu/2020	Yes	Yes	Yes	Yes	Yes	Yes	Yes	Yes	Yes	Yes	N/R	N/R	Yes	Yes	Yes	N/R
10	Barbora Svobodova/2019	Yes	Yes	Yes	Yes	Yes	Yes	Yes	Yes	Yes	N/R	Yes	N/R	Yes	Yes	Yes	N/R
11	Di Hu/2016	Yes	Yes	Yes	no	Yes	Yes	Yes	Yes	Yes	Yes	N/R	N/R	Yes	N/R	Yes	N/R

### Quality assessment

The methodological quality of the included studies was evaluated using the Systematic Review Center for Laboratory Animal Experimentation’s (SYRCLE) risk of bias tool for animal studies. This tool is composed of 10 items reflecting the 6 aspects of the risk of bias: (1) selection bias (sequence generation, baseline characteristics, and allocation concealment); (2) performance bias (random housing, and blinding); (3) detection bias (random outcome assessment, and blinding); (4) attrition bias (incomplete outcome data); (5) reporting bias (selective outcome reporting); and (6) other sources of bias. Indicating “yes” means a low risk of bias, “no” means a high risk of bias, and “N/R” means no sufficient details to measure the risk of bias.

### Ethics approval

This research has been approved by Iran University of Medical Science.

## Results

Our literature search identified a total of 309 articles, of which 72 duplications were excluded. After screening titles and abstracts, 55 records were retrieved for full-text evaluation. Ultimately, 11 studies met predetermined inclusion criteria were included in this systematic review (Table 1).

Table 2 summarizes the basic characteristics of the 11 eligible studies, all published between 2016 and 2022. Animals included Wistar rats in 6 studies (54.5%) [20–25], C57bl/6 mice in 3 studies. (27.2%) [26–28], Sprague Dawley rats in 2 studies (18.18%) [29, 30]. Totally, 495 rats and 137 mice were involved in the eligible studies. The number of animals in each group varied from 7 to 45. The majority of the research investigations focused on the male population, comprising 63.7% of the studies [21, 22, 24–27, 29], while 27.2% of the articles centered on the female demographic [20, 28, 30]. In one particular study, the gender remained unspecified [23]. Among the analyzed research, three investigations were conducted over a 4-week duration [20, 21, 26], two studies spanned 6 weeks [27, 30], and another two studies lasted for 9 weeks [22, 24]. Moreover, two studies took place over a 7-day period [23, 25]; one study was carried out for 8 weeks [29], and a final study reviewed and documented findings up to 12 weeks post-experiment [28].

**Table 2:** Explanation of preclinical study characteristics

	Author/Year	Animal	Gender/ Weight	Animals numbers per group	control group	Intervention	Administration method	Dosage / Wavelengths	Duration of Administration	Duration of study	Purpose of study	Tests and assessments	Outcome
1	Dexiang Ban/2022	Rat / Wistar	Female / 180–250 g	10	Rats with spinal cord injury without treatment	cerium oxide nanoparticles (CONPs)	intrathecal	0.5 mg/mL and 1 mg/mL	one time for 5 min	28 days	investigate the potential of cerium oxide nanoparticles (CONPs) to alleviate neuropathic pain by modulating macrophage polarization.	Mechanical paw withdrawal threshold / Thermal paw withdrawal latency / Immunofluorescence staining / Real-time quantitative polymerase chain reaction	Cerium oxide nanoparticles (CeONPs) alleviate neuropathic pain in a rat spinal cord injury (SCI) model by modulating the polarization of macrophages.
2	Alireza Rahbar/2021	Rat / Wistar	Male / 250-300gr	10	Saline plus multi-walled carbon nanotube (mwcnt)	Ivermectin-functionalized multiwall carbon nanotube	Peritoneally	0.1 mg/kg	3 Continuous days	4 Weeks	Effect of Interactions of Mexiletine with Novel Antiepileptic Drugs in the Maximal Electroshock Test.in this study we tried to compare the effectiveness of IVM combined to MWCNT (IVM-MWCNT) with IVM and minocycline	Basso Beattie and Bresnahan / Hot plate test / Tail-flick latency / Mechanical allodynia - ELISA analysis / Flow cytometry / Oxidative stress	Both IVM-treated and IVM-MWCNT-treated rats had an decrease in spinal cord M1/M2 macrophages relative to control rats, suggesting that these macrophages are possible targets for IVM in neuropathic pain therapy.
3	Fei Chen/2021	Rat / Sprague Dawley	Male / 10-weeks	7	SCI and did not receive any treatment	Isorhamnetin (C16H12O7)	Intraperitoneally	1 and 5 mg/kg/day	Throughout the whole experimental periods	8 Weeks	The aim of current work was to investigate the therapeutic effect of isorhamnetin (ISO) on functional recovery in rats with SCI	Basso Beattie and Bresnahan / Immunohistochemistry / Western blotting analysis / Biochemical analysis / RT-PCR / Cell culture	ISO treatment promoted M2 macrophage activation in the injured region , attenuated neuropathic pain after SCI in rats and promoted functional recovery in rats with SCI by abating oxidative stress and modulating M1/M2 macrophage polarization.
4	Hideaki Nakajima/2020	Mice/C57bl/6	Male/10–12 weeks and 6–8 weeks	45 and 15	Did not receive a spinal cord injury	Bone marrow cells	Intravenously	9.0 Gy	30 min	4 Weeks	Examine differences in the distribution and phenotypes of activated microglia and infiltrated macrophages after SCI at the injured site and lumbar enlargement.	Immunoblot analysis / Flow cytometric analysis / Semi quantitative analysis / Immunohistochemistry / Basso Mouse Locomotor Scale / Dynamic Plantar / Plantar	Activation of M2-type microglia at the lumbar enlargement in response to inflammatory cytokines from the injured site might be important in chronic below-level pain. These findings are useful for establishment of a therapeutic target for prevention of motor deterioration and Neuropathic pain in the time-dependent response to SCI.
5	Jing Zhao/2019	Mice / C57bl/6	Male / N/P	N/R	Sham group (received laminectomy in the absence of contusion)	TMEM16f (Transmembrane protein)	N/R	N/R	N/R	6 Weeks	The potential role of TMEM16F in the progression of SCI	Quantitative real-time PCR / Western blot / Behavior tests / Immunofluorescence / Immunohistochemistry / Cell cultures / Flow cytometry	TMEM16F deficiency regulated the M1/M2 polarization of macrophages/microglia, thus playing important roles in neuropathic pain states following SCI. TMEM16F depletion provided neuroprotection at least partly through changing M1-/M2-like balance towards the M2- like anti neuroinflammatory response
6	Jonghyuck Park/2018	Mice / C57bl/6	Female / 6-8 Weeks	N/R	Sham (laminectomy only), SCI only (without bridge implantation), Bridge alone, Bridge with control virus (firefly-luciferase encoding lentivirus, vCtrl), Bridge with IL-10 lentivirus (vIL-10), and Bridge with IL-4 lentivirus (vIL-4)	IL-10 and IL-4	Implantation of a poly(lactide co-glycolide) (PLG) multichannel bridge	2E9 IU/mL	2 min for absorption into the pores + 3 additional times then	12 Weeks	The impacts of long-term expression of anti-inflammatory cytokines interleukin (IL)-10 or IL-4 on the attenuation of neuropathic pain following SCI	ELISA / RNA isolation and cDNA microarray / Quantitative Reverse-Transcriptase PCR / Behavioral analyses / immunofluorescence	Sustained local expression of anti-inflammatory cytokines by lentivirus delivery after SCI can control macrophage polarization after SCI. Furthermore, aligned configuration of the multichannel bridge, combined with anti-inflammatory cytokines provide synergistic effects to establish appropriate synaptic connections into functional fascicles, resulting in attenuation of chronic neuropathic pain.
7	Arsalan Alizadeh/ 2018	Rat / Sprague Dawley	Female / 8-10 Weeks	40	Uninjured control group	Neuregulin-1	Subcutaneously	2 μg/day	From 3 to 42 days	6 Weeks	How Nrg-1 influences the recruitment and function of immune cells not only within the injured spinal cord tissue but also in the peripheral blood	Flow cytometric / Histological studies / Immunohistochemical / RNA extraction and quantitative real-time PCR	Nrg-1 promotes a pro-regenerative immune response after SCI. Bioavailability of Nrg-1 stimulated a regulatory phenotype in T and B cells and augmented the population of M2 macrophages in the spinal cord and blood after SCI. these data suggest a correlation between downregulation of CXCL1 and reduction in IL-6 expression by Nrg-1 in our acute SCI studies. Notably, inhibition of IL-6 signaling is associated with reduced glial scarring and improves functional recovery and neuropathic pain following SCI.
8	Lucia Machova Urdzikova / 2017	Rat / Wistar	Male / 250-300gr	12	The control group received saline	Epigallocatechin-3-gallate	Directly on the spinal surface and intraperitoneally	50 mg/kg, volume 10 μl and 30μl diluted in saline	Weekly and daily for up to 28 days after SCI .	9 Weeks	Assess if EGCG may regulate inflammatory process in spinal tissue after SCI to improve recovery in rats	Basso Beattie and Bresnahan / Flat beam time score / Flat beam test / Plantar test / Rotarod test / Histological and immunohistochemical / qPCR / Cytokine evaluation	EGCG influenced expression of M1 and M2 macrophage markers, altering the macrophage phenotype and modulating the inflammatory reaction after traumatic SCI. Our results have demonstrated a therapeutic value of EGCG in SCI, as observed by better behavioral performance measured by flat beam test, modulation of inflammatory cytokines and induction of higher axonal sprouting.
9	Di Hu/ 2020	Rat / Wistar	Male / 52±4 Days	40 / 33 / 12 / 11	Sham-injured animals underwent the same surgical procedures without the impaction	Red-Light	Dorsal surface of the animal	670 nm LED; 35 mW/cm2	30 min/day , 7 days	7 Days	The effects of 670 nm treatment during the subacute phase of recovery (prior to 7 dpi) following SCI on: 1) the development of mechanical hypersensitivity; 2) myelination and neuronal apoptosis; and 3) astrocyte and microglia/macrophages presence in the spinal cord and their associated IL-1β and/or iNOS expression in the white matter tracts.	Immunohistochemistry / Sensitivity testing	Red-light treatment significantly reduced the cumulative mechanical sensitivity and the hypersensitivity incidence following SCI. iNOS downregulation may indirectly indicate conversion toward arginase upregulation and therefore conversion toward the M2 phenotype. red-light therapy may represent a useful approach for treating pain during the subacute period after SCI by decreasing neuronal loss and modulating the inflammatory glial response.
10	Barbora Svobodova/2019	Rat / Wistar	Male / 300± 15 g	13	The control group underwent the same treatment without application of the light therapy	Multiwave Locked System	Laser treatment applied to the injury site (t8-t9)	808 nm continuous and 905 nm pulsed	15 minutes after the induction of spinal cord injury for 10 days	9 Weeks	The long term effects and functional recovery after Photobiomodulation (PBM) application.	Basso, Beattie and Bresnahan / Plantar / Beam walk time / Beam walk score / Kinematic analysis / soleus muscle weight / bone strengt / Gray and white matter / Glial scar (GFAP) / Axonal sprouting / immunohistochemical / microglia/ macrophage polarization (cd68 and cd 206) / q-PCR	An upregulation of M2 macrophages in laser treated animals by the increasing number of double labeled CD68+/CD206+ cells in the cranial and central parts of the lesion, compared to the control animals. treatment with PBM suppresses the gene expression of Fgf-2and this could be connected with the significant reduction of hyperalgesia.
11	Di Hu/2016	Rat / Wistar	N/R / 7 Weeks	29	SCI / uninjured group / a sham-injured group / a sham-injured 670-nm-treated group	LED red light	Dorsal surface T10	670 nm	30 min 2 h after surgery and then every 24 h after locomotor assessment for the remainder of the recovery period	7 Days	Evaluate the effect of the 670 nm wavelength following spinal cord injury on a variety of functional parameters, namely the development of hypersensitivity to innocuous stimuli (allodynia), as well as on (tactile) sensory pathway conduction and locomotor recovery, and to see if there were alterations to the M1/M2 sub-populations	Sensitivity assessment / Somatosensory assessment (somatosensory evoked responses, relative response magnitude, latency difference) / Locomotor assessment (contralateral locomotor recovery, ipsilateral locomotor recovery)/ Immunohistochemistry and TUNEL (contralateral tunel, ipsilateral tunel) (ED1 activated monocytes, CD8+ED1+M1 cells, Arginase1+ ED1+ M2 cells)	The proportion of anti-inflammatory/wound-healing (M2) macrophages is greatly enhanced by 24 h following light treatment. enhancing the M2 population during recovery from spinal cord injury may indeed significantly contribute to improving functional outcomes following spinal cord injury. a treatment regime of red light reduces the development of hypersensitivity along with sensorimotor improvements following spinal cord injury.

Table 2 presents the distribution of therapeutic interventions used in the eligible studies, showing that 45.5% involved drug therapy [20–22, 29, 30], 27.2% utilized light therapy interventions, [23–25], 9% pertained to bone marrow transplantation [26], and the remaining 18% examined the impact of cytokines and a specific protein [27, 28] (Table 2).

### Drug therapies

Ban et al. investigated the effects of cerium oxide nanoparticles (CONPs) on neuropathic pain in a rat model of SCI. A total of 30 rats were divided into three groups: the experimental group received intrathecal injection of CONPs, while the control group received intrathecal injection of saline. The study lasted for 4 weeks and found that CONPs significantly increased the number of M2 macrophages in the injured spinal cord of the experimental group compared to the control group. This suggests that CONPs may be able to modulate the M1/M2 macrophage balance in favor of M2 macrophages, which have anti-inflammatory and pro-regenerative properties. The study by Ban et al. provides promising evidence that CONPs may be a new and effective treatment for neuropathic pain after SCI. However, more research is needed to confirm these findings and to determine the optimal dose and delivery method for CONPs [20].

In a study conducted by Rahbar et al. neuropathic pain, macrophage modulation, and oxidative stress were investigated in a rat model of SCI. A total of 40 rats were divided into two groups: the experimental group treated with ivermectin-functionalized multi-walled carbon nanotube (MWCNT) and the control group receiving no treatment. The results demonstrated that the ivermectin-functionalized MWCNTs significantly reduced neuropathic pain in the treated rats compared to the control group. Furthermore, the treatment modulated the M1/M2 macrophage balance and decreased oxidative stress in the injured spinal cord, leading to a better recovery process [21].

A study conducted over a 9-week period by Urdzikova et al. demonstrated that epigallocatechin-3-gallate (EGCG) enhanced neuroregeneration by modulating M2 macrophages and reducing neuroinflammatory cytokine levels, ultimately alleviating neuropathic pain [22].

In a recent study, Chen et al. investigated the potential analgesic effects of isorhamnetin in the context of neuropathic pain for 8 weeks. They divided the rats into a control group and an experimental group, with the latter receiving isorhamnetin treatment. The results demonstrated that isorhamnetin significantly promoted functional recovery in the treated rats by reducing oxidative stress and modulating M2 macrophage/microglia polarization. This suggests that isorhamnetin may be a potential therapeutic agent for SCI recovery by targeting oxidative stress and regulating immune cell polarization [29].

Alizadeh et al. conducted an experiment on mice for a duration of 6 weeks to investigate the effects of Neuregulin-1 (NRG1) administration following SCI. The population included mice with induced SCI, divided into a control group and an intervention group that received NRG1 treatment. The results demonstrated that NRG1 treatment significantly increased the presence of M2 macrophages, which are known to alleviate neuropathic pain, thus suggesting a potential therapeutic role for NRG1 in managing post-SCI neuropathic pain [30].

### Cell transplantation

Nakajima et al. conducted a study in which 60 mice with spinal cord injuries were observed for 4 weeks to analyze the distribution and polarization of microglia and macrophages in injured sites and the lumbar enlargement. The results demonstrated that M2 macrophages played a crucial role in mitigating neuropathic pain and promoting tissue repair [26].

### Cytokines and specific proteins

In a study investigating the effects of transmembrane protein with unknown function 16F (TMEM16F) inhibition on pain-associated behavior and motor function, Zhao et al. administered TMEM16F inhibitors to a group of mice for 6 weeks and compared the results with a control group. They discovered that inhibiting TMEM16F led to a reduction in pain-associated behavior and improved motor function by promoting microglia M2 polarization in mice. This offers a potential therapeutic approach for pain management and motor function improvement [27].

Park and colleagues explored the potential of utilizing lentivirus-mediated delivery of anti-inflammatory cytokines as a means to alleviate inflammation and mitigate neuropathic pain following spinal cord injuries. The experimental design comprised a control group and an intervention group receiving the lentiviral treatment. Spanning 12 weeks, the study's findings revealed a notable decrease in neuropathic pain among the animals in interventional group Furthermore, the treatment fostered the presence of M2 macrophages, which possess anti-inflammatory attributes [28].

### Light therapies

A subsequent investigation conducted by Hu et al. demonstrated the effects of red-light therapy (670 nm) on 85 rats with spinal cord injuries over a 7-day trial. The rats were split into control and intervention groups. The latter group received red-light treatment. Results showed a significant decrease in pain, neuronal cell death, and altered glial responses, indicating that red-light therapy may reduce neuropathic pain by influencing M2 macrophages [25]. Additionally, Hu et al., found that the intervention significantly reduced pain hypersensitivity and improved sensorimotor function, potentially highlighting the role of M2 macrophages in alleviating neuropathic pain [23].

In the final investigation, Svobodova et al. examined the impact of 808 nm and 905 nm wavelength light on recuperation subsequent to SCI over a period of 9 weeks. This study included 26 rats encompassed a control group and an intervention group, the latter of which underwent the light therapy. The findings demonstrated that the employment of 808 nm and 905 nm wavelength light facilitated the activation of M2 macrophages, which contributed substantially to the alleviation of neuropathic pain in the aftermath of SCI [24].

The mentioned investigations have demonstrated promising results in reducing neuropathic pain and functional recovery following SCI through various treatments that modulate macrophage polarization and oxidative stress. For instance, ivermectin-functionalized MWCNTs [21], isorhamnetin [29], and Neuregulin-1 (NRG1) administration [30] have all been found to increase the presence of M2 macrophages, which are known to alleviate neuropathic pain. Other treatment methods, such as TMEM16F inhibition [27] and lentivirus-mediated delivery of anti-inflammatory cytokines [28], also demonstrated a reduction in pain-associated behavior and improved motor function by promoting M2 macrophage polarization. Furthermore, epigallocatechin-3-gallate (EGCG) [22], red-light therapy [25], and 808 nm and 905 nm wavelength light [24] have been shown to enhance neuroregeneration, reduce neuroinflammatory cytokines, and alleviate neuropathic pain by modulating M2 macrophages. These studies suggest that targeting immune cell polarization may offer potential therapeutic approaches for managing neuropathic pain and improving recovery in SCI patients.

## Discussion

M2 macrophages have gained significant attention for their role in modulating inflammation and promoting tissue repair, as well as their potential therapeutic implications [31]. This systematic review is the first in recent years to focus on involvement and significance of M2 macrophages in neuropathic pain following SCI. The review aims to discuss the findings of various studies that have investigated the effects of different treatments and interventions on M2 macrophage modulation and their impact on neuropathic pain management in SCI models.

In summary, this systematic review emphasizes the increasing evidence supporting the involvement and significance of M2 macrophages in neuropathic pain management and recovery after SCI. Various treatments and interventions mentioned in the review illustrate the potential therapeutic advantages of targeting M2 macrophages and modulating their activity to alleviate neuropathic pain and enhance functional recovery in SCI models. Chen et al. illustrated that isorhamnetin treatment promoted the activation of M2 macrophages in the injured area, alleviated neuropathic pain following spinal cord injury (SCI) in rats, and improved functional recovery by diminishing oxidative stress and modulating M1/M2 macrophage polarization. [32]. M2 macrophages are known for their anti-inflammatory effects and tissue repair capabilities, which contribute to alleviating neuropathic pain [29, 32, 33].

Nakajima suggests that M2 phenotype macrophages possess strong anti-inflammatory properties and may be effective in reducing neuropathic pain [26]. One possible way to target M2 macrophages is through anti-inflammatory drugs, which could help to reduce the production of pro-inflammatory cytokines and promote the differentiation of M2 macrophages. Additionally, the role of M2 macrophages is significant in the treatment of neuropathic pain through mesenchymal stem cell (MSC) transplantation [11]. Macrophages play a crucial role in stem cell therapy by creating an immunosuppressive microenvironment that supports the survival and function of transplanted stem cells [34, 35]. Specifically, M2 macrophages have immunosuppressive functions and are responsible for suppressing Th1 immune responses [36]. In addition, macrophages, particularly the M2 type, can affect mesenchymal stem cells (MSCs), and promote MSC proliferation and engraftment [37]. Conversely, MSCs can induce the polarization of M2 macrophages, enhancing their immunomodulatory and tissue repair functions. This reciprocal relationship between macrophages and MSCs is significant in the treatment of neuropathic pain through MSC transplantation [38]. The differentiation of M2 macrophages, facilitated by MSC transplantation, leads to tissue repair, nerve regeneration, modulation of immune responses, and the production of analgesic factors, all contributing to the alleviation of neuropathic pain [39, 40]. The findings can help establish a therapeutic target for preventing motor deterioration and neuropathic pain in the time-dependent response in SCI situation [26].

A potential therapeutic approach for pain management and motor function improvement has been suggested by promoting microglia M2 polarization which can be caused by inhibiting the TMEM16F in mice. TMEM16F deficiency reduces neuropathic pain and modulates M2 macrophages through down-regulating infiltrating macrophages, BAX expression, and pro-inflammatory genes expression. While impeding M1-like activation by reducing the expression of pro-inflammatory markers (TNF-a, IL-1b, IL-18, IL-6, and iNOS) and contributing to elevated expression of M2-anti-inflammatory markers (Ym1, Arg1, SOCS3, and IL-4Ra) [27].

It was also shown that treatment with lentivirus-mediated delivery of anti-inflammatory cytokines (such as interleukin IL-10 and IL-4), shifted immune responses towards pro-regenerative, and increase the number of pro-regenerative M2 macrophages with anti-inflammatory properties, leading to suppressed neuropathic pain, and decreased pro-nociceptive gene expression [28].

Alizadeh et al.'s investigated the effect of neuregulin-1 (Nrg-1) on population of M2 phenotype of macrophages following SCI. Nrg-1 treatment led to a significantly increase in M2 macrophages population (CD45 + CD68 + CD163 + and CD45 + CD68 + CD163 + IL−10 +) in the spinal cord at the acute stage of SCI. M2 macrophages are known to promote oligodendrocyte differentiation, survival, and remyelination, and are associated with overall tissue preservation and improved recovery of function following SCI. The treatment can also modulate the phenotype of macrophages and chemokine profile, leading to a reduction in neuroinflammation and neuropathic pain [30, 41].

It has been reported that epigallocatechin-3-gallate (EGCG), as a major component of green tea, enhanced neuroregeneration after SCI by increasing M2 macrophage cytokines (IL-4, IL-12p70, and TNFα) as well as it reduces the level of neuroinflammatory cytokines and thus can ultimately reduce neuropathic pain. EGCG influences the expression of M1 and M2 macrophage markers, altering the macrophage phenotype and modulating the inflammatory reaction after traumatic SCI [22].

In relation to the role of M2 macrophage, it has been shown that, red-light therapy at 670 nm increases the proportion of M2 macrophages, which can have anti-inflammatory effects and promote tissue repair. The light decreases the proportion of M1 macrophages, which are pro-inflammatory and contribute to tissue damage [37, 42]. This shift towards an M2 phenotype may contribute to the reduction in neuropathic pain observed in the study [25]. Furthermore, Hu et al. discovered that red light treatment significantly influenced the promotion of M2 cell types as early as 24 h post-treatment, leading to reduced cell death and improved sensory and motor functional outcomes. This red light photobiomodulation was found to reduce neuropathic pain and enhance sensorimotor function by decreasing the number of dying cells (TUNEL +) and promoting the expression of the anti-inflammatory (M2) subpopulation (Arginase1 + ED1 +) of macrophages [23, 43].

In the study conducted by Svobodova and colleagues, the impact of 808 nm and 905 nm wavelength light on recovery after SCI was investigated using a multiwave locked system (MLS) laser on rats. The treatment significantly improved locomotor functions, reduced thermal hyperalgesia, and decreased neuropathic pain. The key finding was the upregulation of M2 macrophages in laser-treated animals, evidenced by the increased number of CD68 + /CD206 + cells in the cranial and central parts of the lesion compared to control animals [24]. A shift in microglial/macrophage polarization was confirmed by gene expression analysis, showing significant mRNA downregulation of Cd86 (marker of inflammatory M1 macrophages) and non-significant upregulation of Arg1 (marker of M2 macrophages) [17]. The increase in M2 macrophages and the decrease in M1 macrophages suggest that the MLS laser treatment promotes anti-inflammatory and tissue repair processes, ultimately leading to reduced neuropathic pain and improved functional recovery after SCI [24].

Considering the central role of macrophages in diseases, these cells are considered important targets for the treatment of some diseases [44, 45]. For example, M2 macrophages are targeted for the treatment of gastrointestinal diseases, cancer, bone infections, rheumatoid arthritis, and asthma [46, 47]. By enhancing the anti-inflammatory response, drugs that enhance M2 macrophage polarization may be able to treat various disorders and thereby help patients recover [48, 49].

Sex differences in pain mechanisms are a critical aspect to consider when examining the role of M2 macrophages in neuropathic pain and recovery after spinal cord injury [50]. Evidence suggests that male and female individuals exhibit distinct immune responses, including variations in macrophage activation and cytokine profiles, which can influence pain perception and healing processes [51]. For instance, males tend to exhibit a more pronounced pro-inflammatory response post-injury, potentially leading to heightened pain sensitivity, whereas females often demonstrate a stronger anti-inflammatory response, facilitated by hormones like estrogen [52]. Understanding these sex-specific differences is crucial for developing targeted therapeutic interventions that can more effectively modulate M2 macrophages to alleviate NeP and promote recovery in both sexes.

In addition to SCI, M2 macrophages are relevant in other contexts of NeP, such as peripheral nerve injury, multiple sclerosis, and diabetic neuropathy. These macrophages contribute to reducing inflammation and promoting tissue repair in various nervous system injuries and diseases, thereby mitigating neuropathic pain across different conditions [41].

Further research is needed to optimize these therapeutic approaches and develop effective clinical interventions using M2 macrophages for patients suffering from spinal injuries and neuropathic pain.

## Limitation

Most studies on M2 activation and function are in rats. Therefore, caution must be taken when translating animal studies to humans. Different mouse or rat strains have very different immune and inflammatory responses that differ considerably from humans.

The type and number of macrophages in the injured spinal cord need to be carefully analyzed by studying more specific and better markers.

The prolonged treatment focusing M2 macrophages or regulatory macrophages may have unwanted side effects such as fibrosis, scarring, and tumor progression, in addition to their anti-inflammatory effect.

## Conclusion

In conclusion, this systematic review highlights the involvement and significant of M2 macrophages in neuropathic pain following SCI, particularly in their alleviating role through anti-inflammatory and tissue repair processes. Targeting the regulatory mechanisms of macrophage-driven neuropathic pain could lead to the establishment of novel pharmacotherapies, ultimately improving treatment outcomes for neuropathic pain caused by neuroinflammation following SCI [Supplementary Material 2].

## Supplementary Information

**Additional file 1.** Search Strategies.

## Data Availability

The data that support the findings of this study are available from the corresponding author [Farinaz Nasirinzhad] upon reasonable request.
