# Supplementary material for: The involvement and significance of M2 macrophages in neuropathic pain following spinal cord injury: a systematic review
Source: J Physiol Sci. 2024 Sep 18;74:45. doi: 10.1186/s12576-024-00932-5 (PMC11409760; doi:10.1186/s12576-024-00932-5)
Supplement: Supplementary file 1 — Additional file 1. Search Strategies. [file 12576_2024_932_MOESM1_ESM.docx]

**Supplementary Material 1. Search Strategies**

**MEDLINE (Pubmed)**

("Macrophages"[Mesh] OR "Epithelioid cells"[Mesh] OR "Histiocytes"[Mesh] OR macrophage [All Fields] OR macrophages [All Fields] OR monocyte [All Fields] OR monocyte*[All Fields] OR “epithelioid cell” [All Fields] OR “epithelioid cell*” [All Fields] OR histiocyte[All Fields] OR histiocyte*[All Fields] OR “Langhans giant cells” [All Fields] OR “Foreign Body giant cells” [ALL Fields] OR “Foam Cells” [All Fields]) AND ("Neuralgia"[Mesh] OR "Sciatica"[Mesh] OR "Causalgia"[Mesh] OR hyperalgesia [Mesh] OR Neuralgia [All Fields] OR Neuralgia*[All Fields] OR "Neuropathic Pain"[All Fields] OR "Neuropathic Pain*"[All Fields] OR Neurodynia [All Fields] OR Neurodynia*[All Fields] OR "Nerve Pain"[All Fields] OR "Nerve Pain*"[All Fields] OR Sciatica [All Fields] OR Causalgia [All Fields] OR Hyperalgesia [All Fields] OR Allodynia [All Fields]) AND ("Spinal Cord Injuries"[Mesh] OR "Central Cord Syndrome"[Mesh] OR "Autonomic Dysreflexia"[Mesh] OR "Spinal Cord Compression"[Mesh] OR "Spinal Fractures"[Mesh] OR "Spinal Cord Injuries"[All Fields] OR "Spinal Cord Injury"[All Fields] OR "Central Cord Syndrome"[All Fields] OR "Autonomic Dysreflexia" [All Fields] OR "Spinal Cord Compression"[All Fields] OR "Spinal Cord Compression*"[All Fields] OR "Spinal Fractures"[All Fields] OR "Spinal Fracture"[All Fields] OR "Spinal Cord Trauma"[All Fields] OR "Traumatic Myelopathies"[All Fields] OR Traumatic Myelopathy[All Fields] OR Spinal Cord Laceration[All Fields] OR "Post-Traumatic Myelopathy" [All Fields] OR "Post-Traumatic Myelopathies"[All Fields] OR "Spinal Cord Contusion"[All Fields] OR "Spinal Cord Contusions" [All Fields])

**ISI web of science**

TS=( Macrophage OR "Epithelioid Cell" OR Histiocytes OR monocyte OR “Foam cell” OR “Foreign body giant cell” OR “langhans giant cell”) AND TS=( Neuralgia OR Sciatica OR Causalgia OR "Neuropathic Pain" OR hyperalgesia OR neuropenia OR "Nerve Pain" OR Hyperalgesia OR Allodynia ) AND TS=( “Spinal Cord Injury” OR “Central Cord Syndrome” OR “Autonomic Dysreflexia” OR “Spinal Cord Compression” OR “Spinal Fracture” OR “Spinal Cord Trauma” OR “Traumatic Myelopathy” OR " Spinal Cord Laceration " OR “Post-Traumatic Myelopathy” OR “Spinal Cord Contusion”)

**Scopus**

( TITLE-ABS-KEY ( *"Spinal cord injury"* )  OR  TITLE-ABS-KEY ( *"central cord syndrome"* )  OR  TITLE-ABS-KEY ( *"autonomic dysreflexia"* )  OR  TITLE-ABS-KEY ( *"Spinal Cord Compression"* )  OR  TITLE-ABS-KEY ( *"Spinal Fractures"* )  OR  TITLE-ABS-KEY ( *"Spinal Cord Trauma"* )  OR  TITLE-ABS-KEY ( *"Traumatic Myelopathy"* )  OR  TITLE-ABS-KEY ( *"Spinal Cord Laceration"* )  OR  TITLE-ABS-KEY ( *"Post-Traumatic Myelopathy"* )  OR  TITLE-ABS-KEY ( *"Spinal Cord Contusion"* ) )  AND  ( TITLE-ABS-KEY ( *"Neuropathic pain"* )  OR  TITLE-ABS-KEY ( *neuralgia* ) OR  TITLE-ABS-KEY ( *Hyperalgesia* ) OR  TITLE-ABS-KEY ( *Allodynia* )   OR  TITLE-ABS-KEY ( *sciatica* )  OR  TITLE-ABS-KEY ( *Causalgia* )  OR  TITLE-ABS-KEY ( *Neurodynia* )  OR  TITLE-ABS-KEY ( *"Nerve pain"* )  OR TITLE-ABS-KEY ( hyperalgesia ) OR  TITLE-ABS-KEY ( *allodynia* ) ) AND  ( TITLE-ABS-KEY ( “*Foam cell”* )  OR TITLE-ABS-KEY (“langhans giant cell”) OR TITLE-ABS-KEY ( “*Foreign body giant cell”* )  OR  TITLE-ABS-KEY ( *"Epithelioid cell"* )  OR  TITLE-ABS-KEY ( *histiocyte* )  OR  TITLE-ABS-KEY ( *gliocyte* )  OR TITLE-ABS-KEY ( “*macrophage”* )   TITLE-ABS-KEY ( *monocyte* ) )

**Embase**

(Microglia OR Macrophage OR ‘Epithelioid Cell’ OR Histiocytes OR monocyte OR gliocyte) AND (Neuralgia OR Sciatica OR Causalgia OR ‘Neuropathic Pain’ OR Neurodynia OR ‘Nerve Pain’) OR hyperalgesia OR allodynia) AND (‘Spinal Cord Injury’ OR ‘Central Cord Syndrome’ OR ‘Autonomic Dysreflexia’ OR ‘Spinal Cord Compression’ OR ‘Spinal Fracture’ OR ‘Spinal Cord Trauma’ OR ‘Traumatic Myelopathy’ OR ‘Spinal Cord Laceration‘ OR ‘Post-Traumatic Myelopathy’ OR ‘Spinal Cord Contusion’)
